# Introduction to matrix‐based method for analyzing hybrid multidimensional prostate MRI data

**DOI:** 10.1002/acm2.14544

**Published:** 2024-11-20

**Authors:** Xiaobing Fan, Aritrick Chatterjee, Milica Medved, Tatjana Antic, Aytekin Oto, Gregory S. Karczmar

**Affiliations:** ^1^ Department of Radiology The University of Chicago Chicago Illinois USA; ^2^ Department of Pathology The University of Chicago Chicago Illinois USA

**Keywords:** diffusion‐weighted imaging, eigenvalues, eigenvectors, hybrid multidimensional MRI, matrix, prostate cancer, T2‐weighted imaging

## Abstract

A new approach to analysis of prostate hybrid multidimensional MRI (HM‐MRI) data was introduced in this study. HM‐MRI data were acquired for a combination of a few echo times (TEs) and a few *b*‐values. Naturally, there is a matrix associated with HM‐MRI data for each image pixel. To process the data, we first linearized HM‐MRI data by taking the natural logarithm of the imaging signal intensity. Subsequently, a hybrid symmetric matrix was constructed by multiplying the matrix for each pixel by its own transpose. The eigenvalues for each pixel could then be calculated from the hybrid symmetric matrix. In order to compare eigenvalues between patients, three *b*‐values and three TEs were used, because this was smallest number of *b*‐values and TEs among all patients. The results of eigenvalues were displayed as qualitative color maps for easier visualization. For quantitative analysis, the ratio (*λ*_r_) of eigenvalues (*λ*_1_, *λ*_2_, *λ*_3_) was defined as *λ*_r_ = (*λ*_1_/*λ*_2_)/*λ*_3_ to compare region of interest (ROI) between prostate cancer (PCa) and normal tissue. The results show that the combined eigenvalue maps show PCas clearly and these maps are quite different from apparent diffusion coefficient (ADC) and T2 maps of the same prostate. The PCa has significant larger *λ*_r_, smaller ADC and smaller T2 values than normal prostate tissue (*p* < 0.001). This suggests that the matrix‐based method for analyzing HM‐MRI data provides new information that may be clinically useful. The method is easy to use and could be easily implemented in clinical practice. The eigenvalues are associated with combination of ADC and T2 values, and could aid in the identification and staging of PCa.

## 1 INTRODUCTION

Prostate cancer (PCa) is one of the most common cancers among men.^1^ Accurate assessment of the cancer grade is essential for guiding treatment decisions.^2^ Currently, MRI is considered to be the most effective imaging modality for diagnosis and staging PCa.^3^, ^4^ According to the Prostate Imaging Reporting and Data System version 2.1 (PI‐RADS v2.1), T2‐weighted (T2W) imaging and diffusion‐weighted imaging (DWI) are the two main components of prostate multiparametric MRI (mpMRI).^5^ High‐resolution T2W imaging provides excellent morphological visualization of the gland and accurate prostate anatomy, and is also the dominant sequence for the detection of cancer in transitional zone (TZ).^6^ Apparent diffusion coefficient (ADC) maps obtained from DWI are the preferred sequences for cancer diagnosis in the peripheral zone (PZ).^7^ However, PCa diagnosis with mpMRI still remains problematic as about 15%–30% of clinically significant cancers are missed.^8^, ^9^ In addition, benign features such as benign prostatic hyperplasia (BPH) and prostatitis can mimic PCa on conventional T2W imaging and DWI, and frequently produce false positives.^10^, ^11^, ^12^

Twenty years ago, a hybrid diffusion‐weighted multiple‐echo pulse sequence was used to examine the correlation between the ADC and T2 values of water in rat brain and trigeminal nerve.^13^ This early work showed that trigeminal nerve water ADC changed significantly as a function of TE (echo time). Inspired by this, hybrid multidimensional MRI (HM‐MRI), combining T2 and ADC acquisition was introduced to improve the detection and diagnosis of PCa.^14^ HM‐MRI data are acquired for multiple combinations of TEs and *b*‐values for each image pixel, which is different from conventional mpMRI where T2W and DWI sequences are independently acquired. To optimize sampling, the number of TEs and *b*‐values are often different, for example, with four *b*‐values and three TE values. Previously, HM‐MRI data were initially analyzed based on changes in ADC and T2 as a function of TE and *b*‐value, respectively.^15^ Later, the compartmental model was introduced to analyze HM‐MRI data to determine tissue volume fractions of stroma, epithelium, and lumen.^16^ The dependence of ADC and T2 on TE and *b*‐value, respectively, is very different in cancers versus normal prostate and as well as for PCa with different Gleason Score.^15^ Wang et al. and Sadinski et al. demonstrated that ADC and T2 always changed with increasing TE (increased ADC) and *b*‐value (reduced T2), but did not propose any mathematical model to explain this.^14^, ^15^ Chatterjee et al. showed that fractional volumes of prostatic lumen, stroma, and epithelium measured from HM‐MRI data changed significantly when cancer was present and these fractional volumes could be used as markers to improve the diagnosis of PCa and determine its aggressiveness.^17^ Additionally, the prostate tissue composition measured by using HM‐MRI was very close to that found at quantitative histopathologic evaluation.^17^ These previous studies demonstrated the advantages of using HM‐MRI in the diagnosis of PCa.

HM‐MRI data naturally form a matrix for each pixel, with each element corresponding to image intensity measured at different TEs and *b*‐values. In this study, we introduced a novel method for the analysis of HM‐MRI data by constructing a symmetric matrix and solving for its eigenvalues for each pixel. These eigenvalues are determined by acquired HM‐MRI data, provide a simple way to represent the changes in ADC and T2 as a function of TE and *b*‐value. Our proposed matrix analysis explores difference in how signal changes with imaging parameters for different tissue, denoted by these eigenvalues for cancer diagnosis. Therefore, we investigated whether ‘MAHM’ (Matrix Analysis of Hybrid MRI) has the potential to aid the diagnosis of PCa.

## 2 METHODS

### 2.1 Theory

HM‐MRI data are normally acquired with *M* different TEs and *N* different *b*‐values. Consequently, for each pixel an *M *× *N* real matrix ***A*** is obtained, with each entry representing imaging signal intensity acquired with a specific combination of TE and *b*‐value. The matrix ***A*** is frequently not a square matrix due to different numbers of TE and *b*‐value used in protocol and therefore cannot be calculated for eigenvalues. Even if it is a square matrix, the eigenvalues are not necessarily real numbers. Only a symmetric matrix with real entries has all real eigenvalues and orthogonal eigenvectors.^18^ Therefore, a symmetric matrix must be generated from HM‐MRI data in order to calculate its eigenvalues.

We define $H\;=\ln A\times {\left(\ln A\right)}^{T}$ or $H\;={\left(\ln A\right)}^{T}\times \ln A$, whichever results in the smaller size of the resulting matrix, where lnA consists of natural logarithms of the corresponding elements of ***A***, and ${\left(\ln A\right)}^{T}$ is transpose of lnA. We linearize the data using logarithms because the imaging signal intensity is approximately exponentially dependent on both TEs and *b*‐values. Thus, ***H*** is a real symmetric matrix because it is a square matrix whose transpose is equal to the matrix itself.^18^ Therefore, we can calculate its eigenvalues (*λ*_1 _>* λ*_2 _> *λ*_3 _> …) and corresponding eigenvectors (${\vec{e}}_{1}$, ${\vec{e}}_{2}$, ${\vec{e}}_{3}$, …) easily using commercial software. For each slice, eigenvalues could be used to generate color maps for qualitative evaluation of HM‐MRI. The ratio of eigenvalues could be used for qualitative analysis by radiologists where all the information is combined in one image.

As an example of the matrix analysis method, we assume HM‐MRI data acquired using three *b*‐values (*b*_1_ < *b*_2_ < *b*_2_) for each of two TEs (TE_1_ < TE_2_). Then, there is a matrix ***A*_2×3_** associated with different *b*‐values and different TEs for each pixel, that is,

(1) $$\begin{array}{ccc} & & A_{2\times 3}=\left[\begin{array}{ccc} a_{11} & a_{12} & a_{13}\\ a_{21} & a_{22} & a_{23} \end{array}\right]=\\ & & \left[\begin{array}{ccc} A_{1}e^{-TE_{1}/T_{2}}\cdot e^{-b_{1}D} & A_{1}e^{-TE_{1}/T_{2}}\cdot e^{-b_{2}D} & A_{1}e^{-TE_{1}/T_{2}}\cdot e^{-b_{3}D}\\ A_{2}e^{-TE_{2}/T_{2}}\cdot e^{-b_{1}D} & A_{2}e^{-TE_{2}/T_{2}}\cdot e^{-b_{2}D} & A_{2}e^{-TE_{2}/T_{2}}\cdot e^{-b_{3}D} \end{array}\right]\;\;, \end{array}$$

where $a_{ij}$ (i = 1, 2; j = 1, 2, 3) is entry of matrix, *A*_i_ (i = 1, 2) is a proportionality constant, T2 is the transverse relaxation time, and D is the ADC.^19^ Then the following symmetric hybrid matrix ***H*** can be generated:

(2) $$\begin{array}{ccc} H_{2\times 2} & = & \left[\begin{array}{cc} H_{11} & H_{12}\\ H_{21} & H_{22} \end{array}\right]=\ln A\times {\left(\ln A\right)}^{T}\\ & = & \left[\begin{array}{cc} {\left(\ln a_{11}\right)}^{2}+{\left(\ln a_{12}\right)}^{2}+{\left(\ln a_{13}\right)}^{2} & \ln a_{11}\cdot \ln a_{21}+\ln a_{12}\cdot \ln a_{22}+\ln a_{13}\cdot \ln a_{23}\\ \ln a_{11}\cdot \ln a_{21}+\ln a_{12}\cdot \ln a_{22}+\ln a_{13}\cdot \ln a_{23} & {\left(\ln a_{21}\right)}^{2}+{\left(\ln a_{22}\right)}^{2}+{\left(\ln a_{23}\right)}^{2} \end{array}\right]. \end{array}$$

The eigenvalues and eigenvectors of matrix ***H_2__×__2_*** can be calculated easily. Two eigenvalues *λ*_i_ (i = 1, 2) are $\lambda_{1,2}=\frac{1}{2}\;\left(\mathrm{tr}\left(H\right)\pm \sqrt{{\left[\mathrm{tr}(H)\right]}^{2}-4\det\left(H\right)}\right)$, and approximately equal to:

(3) $$\lambda_{1,2}\approx \left\{\begin{array}{c} tr\left(H\right)-\det\left(H\right)/tr\left(H\right),\\ \det\left(H\right)/tr\left(H\right). \end{array}\right.$$

where tr(H) and det(H) are the trace and determinant of matrix ***H***. The corresponding eigenvectors are equal to:

(4) $$\;{\vec{e}}_{1,2}=\left[\begin{array}{c} H_{12}\\ \lambda_{1,2}-H_{11} \end{array}\right]\;\;\mathrm{or}\;\;{\vec{e}}_{1,2}=\left[\begin{array}{c} \lambda_{1,2}-H_{22}\\ H_{21} \end{array}\right]\;.$$

For any 3 × 3 or higher order matrix, eigenvalues can be easily calculated using commercial software, which avoids the need for curve‐fitting such as in calculating ADC and T2 values.^20^

### 2.2 MRI data acquisition

Total *n* = 18 patients with histologically confirmed PCa were included in this study. All patients provided prior informed written consent, which was compliant with the Health Insurance Portability and Accountability Act (HIPAA).

HM‐MRI data were acquired on a Philips Achieva 3T‐TX scanner (Philips Healthcare, Netherlands). The hybrid protocol composed of a single spin‐echo module with diffusion sensitizing gradients placed symmetrically around the 180‐degree pulse, followed by single‐shot EPI read‐out.^14^ HM‐MRI data were acquired with three or four TEs between 47–200 ms. For each TE, images were acquired with three or four *b*‐values between 0 ‐ 1500 s/mm^2^ (TR = 3500 ms, field of view = 180 × 180 mm^2^, matrix size = 72 × 72, reconstruction matrix = 128 × 128, slice thickness = 3 mm, number of slices = 18). The acquisition time was 10–12 mins. The detailed number of *b*‐values and TEs used for data acquisition is given in Table 1. In addition, mpMRI data with the standard clinical protocol, including T2W imaging, DWI, and dynamic contrast enhanced (DCE) imaging were also acquired for the diagnosis of PCa.

**TABLE 1:** The TEs and *b*‐values used for HM‐MRI data acquisition.

No. of Patient	TEs (ms)	*b*‐values (s/mm^2^)
**1**	47, 75, 100	0, 750, 1500
**1**	70, 150, 200	0, 150^a^, 1000, 1500
**14**	57, 80, 150, 200^a^	0, 150^a^, 1000, 1500
**2**	57, 80, 150, 200^a^	0, 150^a^, 1000, 1500

Abbreviations: HM‐MRI, hybrid multidimensional MRI; TEs, echo times.

^a^ Removed *b*‐values or TEs for those patients acquired with more than three so that all patients have the same number of *b*‐values and TEs.

### 2.3 Histology

After MRI, the patients underwent radical prostatectomy. The prostatectomy specimen was sectioned (4 mm thick sections) approximately in the same plane as MR images and H&E stained. PCa lesions were graded and outlined by an experienced pathologist (T.A. ‐ 18 years’ experience). H&E stained whole mount radical prostatectomy sections were matched with corresponding prostate MR images by an expert radiologist (A.O. ‐ 20 years’ experience with prostate MRI). One cancer and one normal tissue region of interest (ROI) from the contralateral side were traced for each patient based on H&E slices and closely matched ADC and T2 maps.

### 2.4 Data analysis

MRI data were analyzed using IDL 8.8.1 (Harris Geospatial Solutions, Inc. CO, USA) with an in‐house software package. Since smallest number *b*‐values and TEs was three among all 18 patients, we had to remove *b*‐values or TEs for those patients acquired with more than three so that all patients had the same number of eigenvalues for comparison (Table 1). The remaining *b*‐values or TEs should be close to the case acquired with three *b*‐values and three TEs. Then matrix analysis was performed on a pixel‐by‐pixel basis using the aforementioned method described above.

For qualitative analysis, three primary colors, Red, Green, and Blue (RGB) were assigned to the first, second, and third eigenvalues and the lightness of each color was used to represent the corresponding eigenvalue. For the best visual effect, zero and the median eigenvalue over the whole prostate were used as the minimum and maximum values to scale the values to 0 and 255 for each color display eigenvalue map. Therefore, at each pixel the value for each color is an 8‐bit number, in the range 0–255. The final eigenvalue map was displayed as merged RGB images using the three separate channels, that is, a new RGB stack was created.

Because the signal intensities of HM‐MRI data vary between patients due to different *b*‐values and TEs used in data acquisition, we cannot directly compare eigenvalues between patients. Therefore, for quantitative analysis, the ratio (*λ*_r_) of eigenvalues (*λ*_1_, *λ*_2_, *λ*_3_) was introduced *λ*_r_ = (*λ*_1_/*λ*_2_)/*λ*_3_ to compare ROI between PCa and normal tissue. Apparently, the ratio provides some degree of normalization.

Maps of ADC and T2 were also generated from HM‐MRI data. The ADC in each pixel was calculated by fitting the raw data using the following equation^20^:

(5) $$\;S_{b}=S_{\mathrm{SE}}\;\exp\left(-b\cdot D\right),$$

where S_b_ is the attenuated spin‐echo signal and S_SE_ is the maximum spin‐echo signal without diffusion attenuation. T2 was calculated by fitting the raw data with the equation^20^:

(6) $$\;S_{\mathrm{TE}}=S_{0}\;\exp\left(-\mathrm{TE}/T_{2}\right),$$

where S_TE_ is signal measured at each TE and S_0_ is the extrapolated signal at TE = 0. The matrix analysis results were also qualitatively compared with T2 maps and ADC maps calculated from HM‐MRI data.

### 2.5 Statistical analysis

The Wilcoxon signed‐rank test was performed to determine whether there was significant difference between cancer and normal prostate tissue for *λ*_r_, ADC, and T2. A *p*‐value less than 0.05 was considered statistically significant. Receiver operating characteristic (ROC) analysis was used to evaluate performance of *λ*_r_, ADC, and T2 in differentiating cancer from normal tissue.

## 3 RESULTS

**FIGURE 1:**
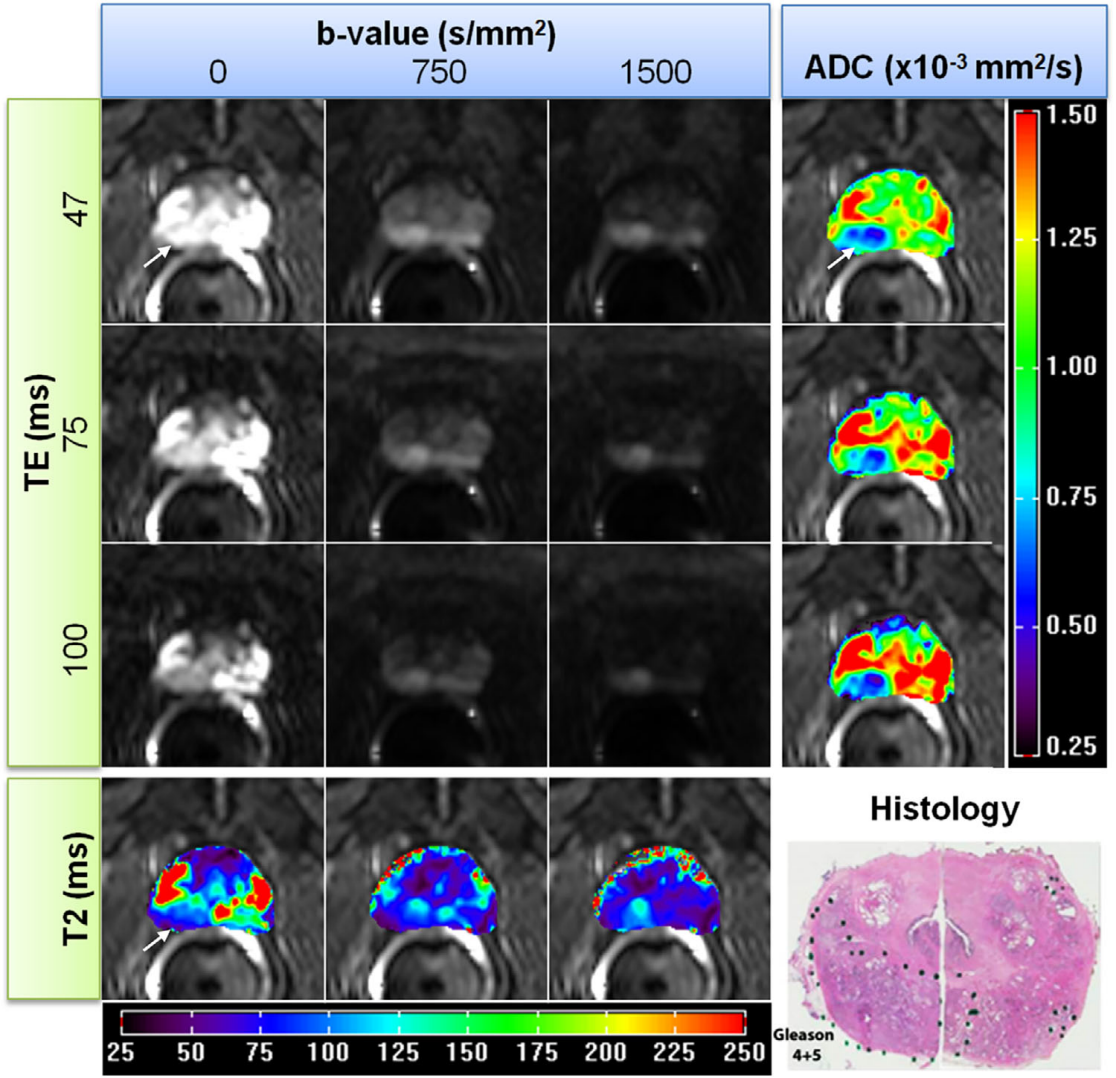
Top left panel shows an example of HM‐MR images (gray) acquired for 60‐year‐old patient with three different *b*‐values and three different TEs for a selected slice with prostate cancer. The gray images were displayed in the same scale to show the intensity changes as a function of different *b*‐values and TEs. The corresponding color T2 maps (most‐bottom row) and color ADC maps (most‐right column) calculated from these hybrid data as a function of *b*‐values and TEs were superimposed over hybrid image with the smallest TE and 0 *b*‐value. The maps were obtained by fitting the data with the exponential decay function using Equations (5) and (6). A corresponding histology slice is shown at lower right corner of the panel. The cancer is indicated by white arrow. ADC, apparent diffusion coefficient; HM‐MRI, hybrid multidimensional MRI; PCa, prostate cancer; TEs, echo times.

For a selected HM‐MRI slice from a 60‐year‐old patient, Figure 1 (top left panel) shows an example of HM‐MR images (gray) with three *b*‐values (left, middle, and right column) for each of three TEs (top, middle, and bottom row) displayed at the same gray scale. Thus, there is a 3 × 3 hybrid matrix of image intensity values for each pixel. As expected, the imaging signal intensity decreases as TE and *b*‐value increase. The corresponding prostate ADC color maps (most‐right column) for each TE and T2 color maps (most‐bottom row) for each *b*‐value were calculated from the hybrid data using Equations 5 and 6 and superimposed on the image with shortest TE and 0 *b*‐value. As we can see, changes in ADC maps as a function of TE, and in T2 maps as a function of *b*‐value are evident. The cancer in the (viewers) right PZ (shown on histology in the lowest right corner) is shown much more clearly in the higher TE and *b*‐value hybrid image, especially in the ADC map calculated from higher TE.

**FIGURE 2:**
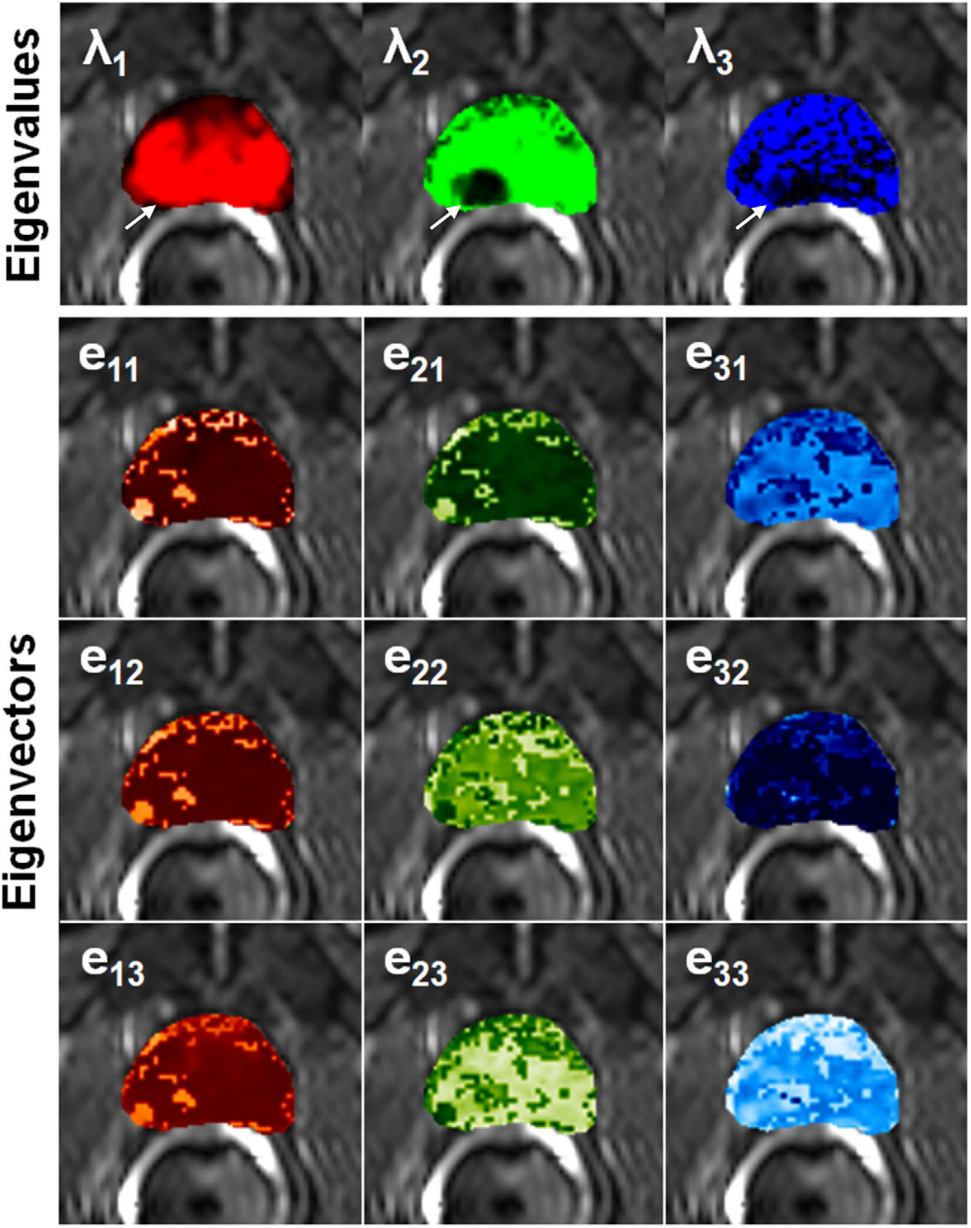
For the same data shown in Figure 1, the eigenvalues and eigenvectors for this 3 × 3 matrix were calculated by using MAHM for each pixel. The eigenvalue color maps (top row panel) were displayed in RGB from left to right for eigenvalue *λ*_1_, *λ*_2_, and *λ*_3_, respectively. The corresponding eigenvectors ${\vec{e}}_{1}$(e_11_, e_12_, e_13_), ${\vec{e}}_{2}$(e_21_, e_22_, e_23_), and ${\vec{e}}_{3}$(e_31_, e_32_, e_33_) components color maps were shown in columns underneath each eigenvalue. Since the calculated eigenvectors are normalized to have unit length eigenvectors, ‐1 and 1 were used as the minimum and maximum values to scale the values to 0 and 255 for each color display eigenvectors components maps. All color maps were superimposed over hybrid image with smallest TE and 0 *b*‐value. The cancer is indicated by white arrow. MAHM, Matrix Analysis of Hybrid MRI; RGB, red, blue, and green; TE, echo time.

For the same slice shown in Figures 1, Figure 2 top row shows RGB (left, middle, and right) eigenvalue color maps calculated with our matrix analysis technique: *λ*_1_ (red), *λ*_2_ (green), and *λ*_3_ (blue). The PCa in right PZ is hyperintense on *λ*_1_ (red) map and hypointense on *λ*_2_ (green) and *λ*_3_ (blue) maps, compared to benign tissue. As an example, the corresponding eigenvectors components color maps are shown in the column underneath of each eigenvalue map: ${\vec{e}}_{1}$(e_11_, e_12_, e_13_) (left), ${\vec{e}}_{2}$(e_21_, e_22_, e_23_) (middle), and ${\vec{e}}_{3}$(e_31_, e_32_, e_33_) (right). All color maps were superimposed on the image with the shortest TE and 0 *b*‐value. These color maps clearly show differences between individual eigenvalues and eigenvectors components.

**FIGURE 3:**
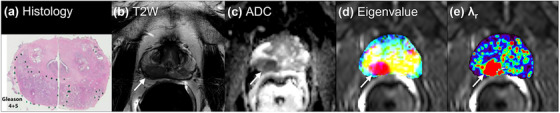
For the same 60‐year‐old patient shown in Figure 1, results of MAHM: (a) whole‐mount histology from prostatectomy specimen with cancer markers, (b) corresponding T2W high‐resolution image, (c) ADC map, (d) merged three color eigenvalues maps, and (e) rainbow color *λ*_r_ map superimposed over hybrid image with smallest TE and 0 *b*‐value. The red color region shows cancer in the merged color eigenvalue map and the rainbow color *λ*_r_ map. The cancer is indicated by white arrow. ADC, apparent diffusion coefficient; MAHM, Matrix Analysis of Hybrid MRI; T2W, T2‐weightedTE, echo time.

For the same patient shown above, Figure 3 shows (a) whole‐mount histology with Gleason 4 + 5 right posterolateral cancer and other two small cancers (circled with dots), (b) high‐resolution T2W image, (c) ADC map, (d) final eigenvalue map (merged three color maps) with red regions indicating cancers, and (e) rainbow color *λ*_r_ map with red regions indicating cancers. The merged eigenvalue map and the *λ*_r_ map show the cancers clearly, and is very different from the ADC and T2 maps shown in Figure 1. This suggests that MAHM is providing new and useful information. Importantly, anterior fibromuscular stroma (AFMS) appears similar to benign tissue on the eigenvalue maps, despite having low T2 (at *b* = 0 s/mm^2^) and ADC (at TE = 100 ms) values mimicking PCa on T2 and ADC maps.

**FIGURE 4:**
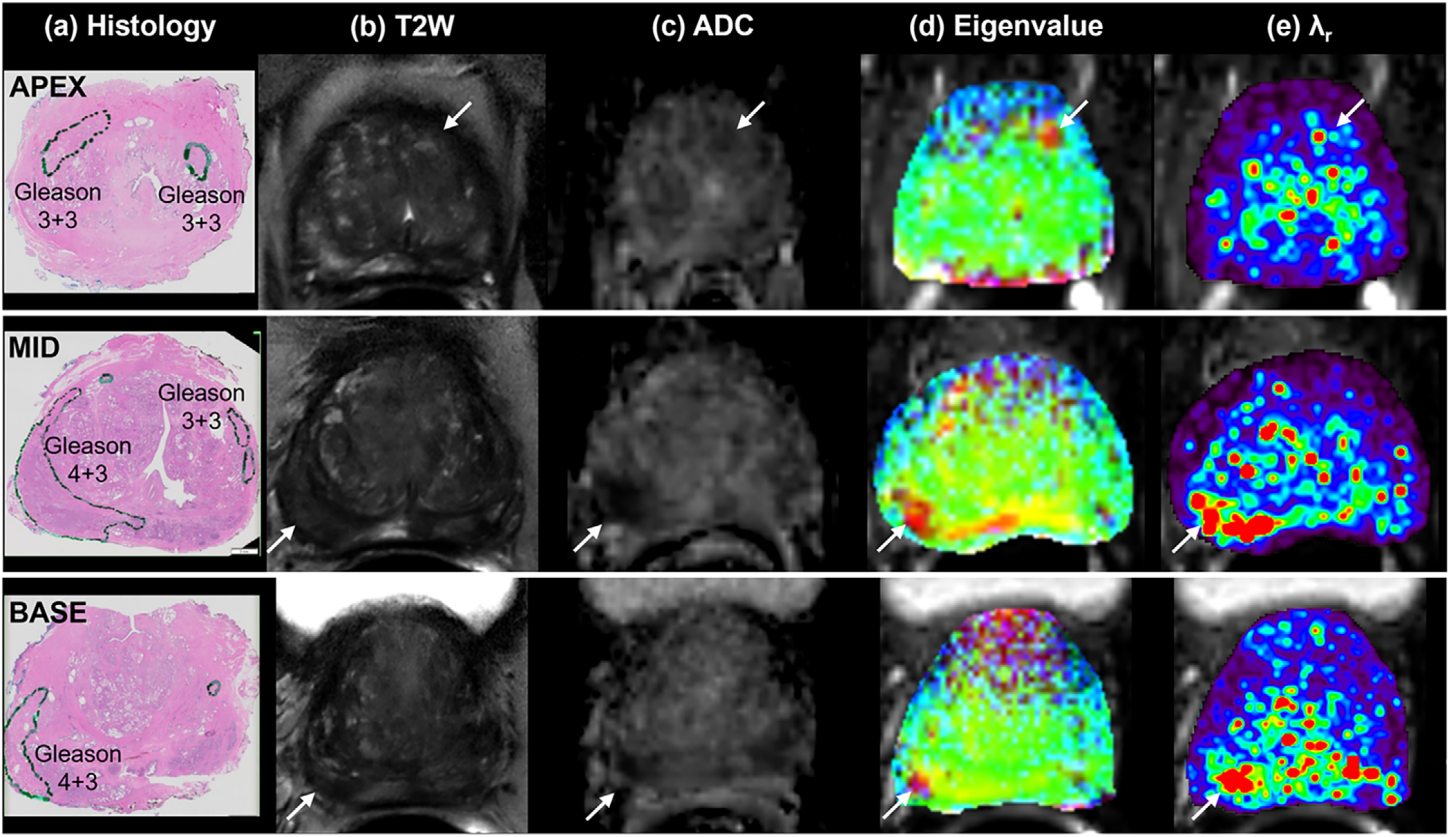
Results of MAHM for 62‐year‐old patient show at three slices near apex (top row), mid (middle row), and base (bottom row) of prostate: (a) whole‐mount histology from prostatectomy specimen with cancer markers, (b) corresponding T2W high‐resolution image, (c) ADC map, (d) merged three color eigenvalues maps, and (e) rainbow color *λ*_r_ map superimposed over hybrid image with smallest TE and 0 *b*‐value. The cancer is indicated by white arrow. ADC, apparent diffusion coefficient; T2W, T2‐weighted; TE, echo time.

Figure 4 shows another example of MAHM for a 62‐year‐old patient with three slices near apex (top row), mid (middle row), and base (bottom row) of prostate. The corresponding whole‐mount histology, high‐resolution T2W image, ADC map, merged eigenvalue map, and rainbow color *λ*_r_ map are shown in column (a—e) from left to right, respectively. Again, the merged eigenvalue map and the *λ*_r_ map show cancer in multiple slices. The right posterolateral Gleason 4 + 3 cancer in the mid and base section are clearly visible as red on the eigenvalue map and the *λ*_r_ map. But Gleason 3 + 3 cancers are not highlighted in the eigenvalue maps and the *λ*_r_ maps.

**FIGURE 5:**
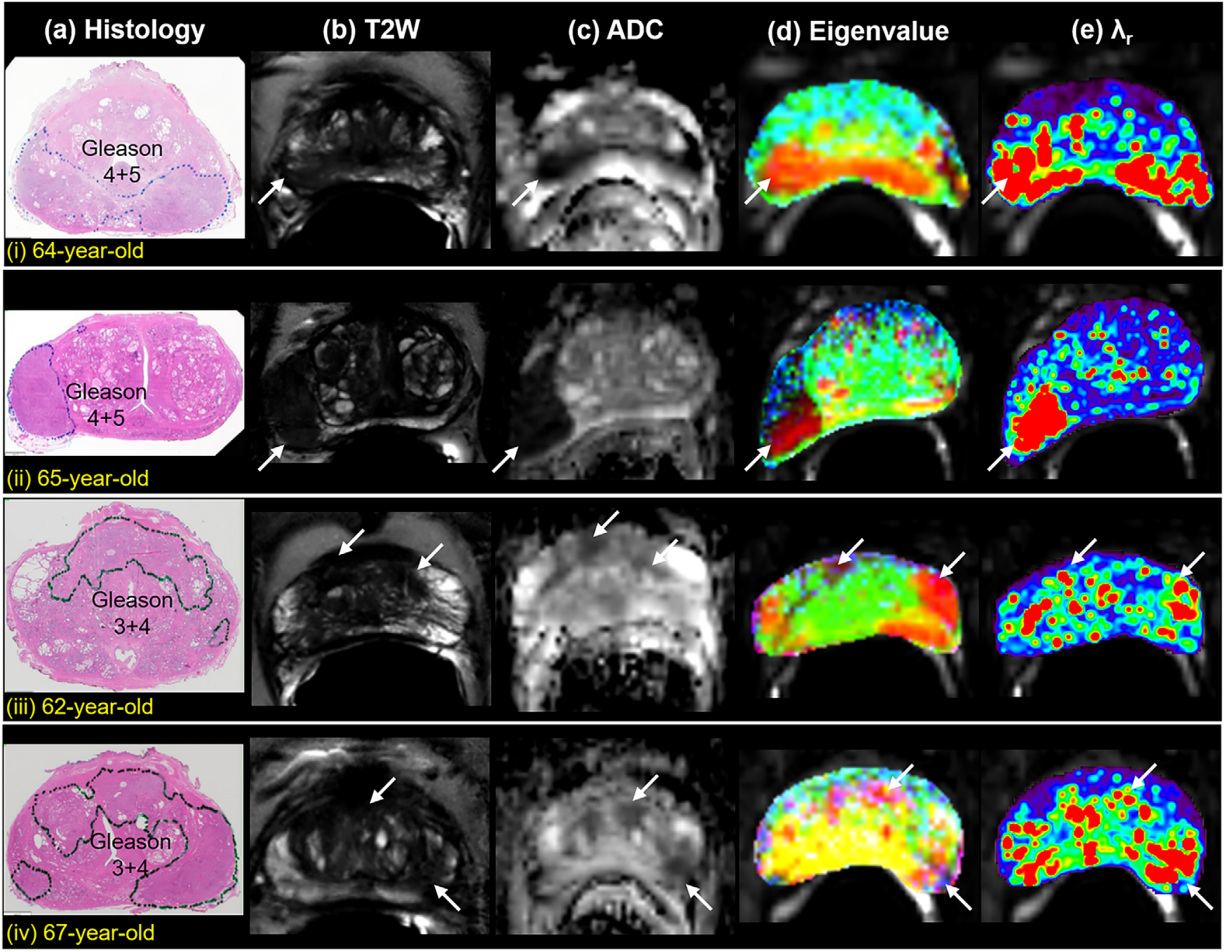
Results of MAHM show for (i) 64‐year‐old patient with Gleason 4 + 5 cancer in the PZ, (ii) 65‐year‐old patient with Gleason 4 + 5 cancer in the right PZ, (iii) 62‐year‐old patient with Gleason 3 + 4 anterior cancer, and (iv) 67‐year‐old patient with Gleason 3 + 4 cancer: (a) whole‐mount histology with cancer markers, (b) corresponding T2W image, (c) ADC map, (d) merged three color eigenvalues maps, and (e) rainbow color *λ*_r_ map superimposed over hybrid image with smallest TE and 0 *b*‐value. The cancer is indicated by white arrow. ADC, apparent diffusion coefficient; MAHM, Matrix Analysis of Hybrid MRI; PZ, peripheral zone; T2W, T2‐weightedTE, echo time.

Figure 5 shows MAHM results for four more patients (top to bottom row i—iv): (a) whole‐mount histology, (b) high‐resolution T2W image, (c) ADC map, (d) merged color eigenvalue map, and (e) rainbow color *λ*_r_ map. The results suggest that MAHM could aid in the diagnosis of PCa.

**FIGURE 6:**
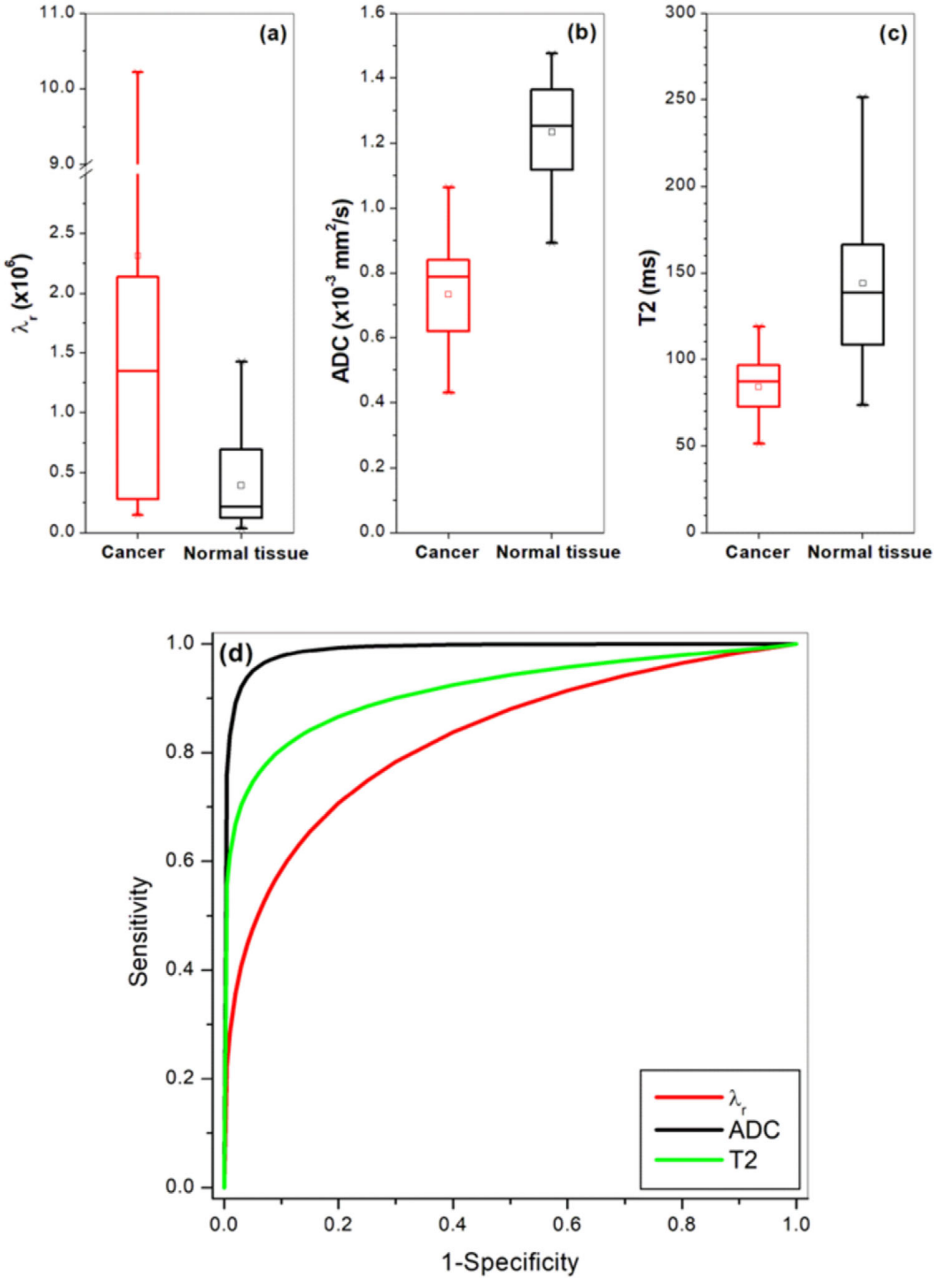
Box‐plots of (a) *λ*_r_, (b) ADC, and (c) T2 for comparisons between cancer and normal tissue. Plot of AUC (d) obtained from ROC analysis results for *λ*_r_, ADC, and T2 in differentiating cancer from normal tissue. The square (□) indicates mean and the asterisks (*) indicate the upper and lower limits of the data. ADC, apparent diffusion coefficient; AUC, area under the curve; ROC, receiver operating characteristic.

Finally, Figure 6a–c shows the box‐plots of (a) *λ*_r_, (b) ADC, and (c) T2 for comparison between cancer and normal tissue. The Wilcoxon signed‐rank test showed that there was a statistically significant difference (*p* < 0.001) between cancer and normal tissue for *λ*_r_, ADC, and T2. Figure 6d shows ROC analysis results for *λ*_r_, ADC, and T2 in differentiating between cancer and normal tissue with area under the curve (AUC) of 0.83, 0.99, and 0.92, respectively. Since traced ROIs were biased for ADC and T2, as radiologists used ADC maps and T2W to identify cancer location after rad‐path correlation, it is not surprising that ADC and T2 had higher AUC values. On the other hand, *λ*_r_ suffers from variations in MRI signal intensities due to motion and noise at different *b*‐values and TEs used in data acquisition and caused lower AUC value, even though paired statistical test shows significant difference between cancer and normal tissue. However, combining *λ*_r_ with T2 and ADC gives an AUC of 1.00.

## 4 DISCUSSION

The proposed matrix‐based method is a new and promising way to analyze HM‐MRI data and aid in detecting PCa. MAHM is easy to apply and could be used clinically. Our preliminary results shown here suggest that PCa can be seen clearly on eigenvalue maps. These maps are visually different from ADC maps and T2 maps calculated from HM‐MRI data. The eigenvalue ratio *λ*_r_ is a statistically significant difference between cancer and normal tissue. There is an advantage to using the ratio *λ*_r_ instead of individual eigenvalue because it can be directly compared between patients if HM‐MRI data were acquired with the same *b*‐values and TEs. Therefore, it is likely that MAHM data provide new information and may be useful for the diagnosis of PCa.

Potential advantages of MAHM data include: (i) the analysis is model‐free and may provide information that is not available from compartmental models; (ii) a real symmetric hybrid matrix (***H***) can be constructed even if acquired HM‐MRI matrix data are not square; (iii) eigenvalues can be quickly and reliably calculated; (iv) maps generated with eigenvalues reflect the tissue properties associated with T2 at different *b*‐values and ADC at different TEs, but efficiently combine all HM‐MRI data.

Although the primary purpose of the work discussed here is to introduce a new technique for analysis of HM‐MRI data, the results show the diagnostic potential of the method. The merged eigenvalue map shows cancers very clearly—including in some cases where the cancer is not easily seen on the T2W image and ADC maps (Figure 4 and 5 first row). Since eigenvalue maps are very different from ADC and T2 maps, combinations of all of these parameters may increase diagnostic accuracy. Despite the fact that matrix eigenvalues can be easily and quickly calculated by using commercial software, the noise of HM‐MRI data could have a serious impact on calculated eigenvalues. We believe this is partially the reason why some cancers are not clearly shown in the eigenvalue maps and the size of cancer is underestimated compared with ADC and T2 maps. This is a disadvantage of matrix analysis compared to analyzing data by using the curving fitting, which can involve either interpolation and/or smooth data guided by used mathematical model.

There are several limitations of this study. First, the sample size is relatively small and *b*‐values and TEs used in data acquisition are not the same for all patients. Evaluation of a large number of cases in multi‐center setting is needed to determine whether matrix‐based method complements or is more effective than other techniques for analyzing HM‐MRI data. Second, the merged eigenvalue map significantly underestimates tumor size—based on comparison with histology. This can be corrected when we have more data to use logistic regression or related methods to produce an optimal combination parameters and cutoffs. Third, the accuracy of matrix analysis may be affected by motion artifacts because DWI is very sensitive to motion. Improved data acquisition and/or filtering may be needed prior to MAHM. For example, methods previously developed to identify and correct DWI data that is corrupted by motion could be used to improve diagnostic accuracy.^21^, ^22^ Finally, sampling of *b*‐values and TE values may not be optimal—more evenly distributed *b*‐values and TE values could be beneficial, especially to avoid extreme larger b‐value and longer TE to produce noise data.

## 5 CONCLUSION

Since the dependence of ADC and T2 as function of TE and *b*‐value is different for cancers versus normal prostate,^14^ it is likely that the eigenvalues isolate the cancer signals from normal tissue. MAHM is an effective new method for analyzing HM‐MRI data to capture these differences. Therefore, maps of individual eigenvalues and the merged eigenvalue map obtained from matrix analysis could help in aiding the identification of PCa. The ratio (*λ*_r_) of eigenvalues was significantly higher for PCa than normal tissue. MAHM could also be applied to HM‐MRI data from other tissues and organs, and to other multidimensional MR data.

## AUTHOR CONTRIBUTIONS

Xiaobing Fan conceived the concept, analyzed the data, wrote the draft, and edited the final manuscript. Aritrick Chatterjee acquired and analyzed the data and edited the manuscript. Milica Medved edited the manuscript and provided critical input. Tatjana Antic analyzed the data. Aytekin Oto supervised the study and contributed to the content of the manuscript. Gregory S. Karczmar supervised the study, contributed to the content of the manuscript, and edited the final manuscript. All authors have read and agreed to the published version of the manuscript.

## CONFLICT OF INTEREST STATEMENT

Aritrick Chatterjee, Aytekin Oto, and Gregory S. Karczmar report equity in QMIS LLC, outside the submitted work. The other authors have no relevant financial or non‐financial interests to disclose.

## ETHICS STATEMENT

The study was approved by the Ethics Committee of our institute—Institutional Review Boards (IRB) (IRB13‐0756).

## References

[1] Crawford ED . Epidemiology of prostate cancer. Urology. 2003;62(suppl 1):3‐12. 10.1016/j.urology.2003.10.013 14706503

[2] Chen RC , Rumble RB , Loblaw DA , et al. Active surveillance for the management of localized prostate cancer (Cancer Care Ontario Guideline): American Society of Clinical Oncology Clinical Practice Guideline Endorsement. J Clin Oncol. 2016;34(18):2182‐2190. 26884580 10.1200/JCO.2015.65.7759

[3] Ahmed HU , El‐Shater Bosaily A , Brown LC , et al. Diagnostic accuracy of multi‐parametric MRI and TRUS biopsy in prostate cancer (PROMIS): a paired validating confirmatory study. Lancet. 2017;389(10071):815‐822. 28110982 10.1016/S0140-6736(16)32401-1

[4] Brown LC , Ahmed HU , Faria R , et al. Multiparametric MRI to improve detection of prostate cancer compared with transrectal ultrasound‐guided prostate biopsy alone: the PROMIS study. Health Technol Assess. 2018;22(39):1‐176. 10.3310/hta22390 PMC6077599 30040065

[5] Turkbey B , Rosenkrantz AB , Haider MA , et al. Prostate Imaging reporting and data system version 2.1: 2019 update of prostate imaging reporting and data system version 2. Eur Urol. 2019;76(3):340‐351. 30898406 10.1016/j.eururo.2019.02.033

[6] Krishna S , Schieda N , McInnes MD , Flood TA , Thornhill RE . Diagnosis of transition zone prostate cancer using T2‐weighted (T2W) MRI: comparison of subjective features and quantitative shape analysis. Eur Radiol. 2019;29(3):1133‐1143. 30105411 10.1007/s00330-018-5664-z

[7] Tamada T , Sone T , Jo Y , et al. Apparent diffusion coefficient values in peripheral and transition zones of the prostate: comparison between normal and malignant prostatic tissues and correlation with histologic grade. J Magn Reson Imaging. 2008;28(3):720‐726. 18777532 10.1002/jmri.21503

[8] Borofsky S , George AK , Gaur S , et al. What are we missing? False‐negative cancers at multiparametric MR imaging of the prostate. Radiology. 2018;286(1):186‐195. 29053402 10.1148/radiol.2017152877 PMC5749595

[9] Fütterer JJ , Briganti A , De Visschere P , et al. Can Clinically significant prostate cancer be detected with multiparametric magnetic resonance imaging? A systematic review of the literature. Eur Urol. 2015;68(6):1045‐1053. 25656808 10.1016/j.eururo.2015.01.013

[10] Kitzing YX , Prando A , Varol C , Karczmar GS , Maclean F , Oto A . Benign conditions that mimic prostate carcinoma: MR imaging features with histopathologic correlation. Radiographics. 2016;36(1):162‐175. 26587887 10.1148/rg.2016150030 PMC5496681

[11] Oto A , Kayhan A , Jiang Y , et al. Prostate cancer: differentiation of central gland cancer from benign prostatic hyperplasia by using diffusion‐weighted and dynamic contrast‐enhanced MR imaging. Radiology. 2010;257(3):715‐723. 20843992 10.1148/radiol.10100021 PMC6939960

[12] Schiebler ML , Tomaszewski JE , Bezzi M , et al. Prostatic carcinoma and benign prostatic hyperplasia: correlation of high‐resolution MR and histopathologic findings. Radiology. 1989;172(1):131‐137. 2472644 10.1148/radiology.172.1.2472644

[13] Does MD , Gore JC . Compartmental study of diffusion and relaxation measured in vivo in normal and ischemic rat brain and trigeminal nerve. Magn Reson Med. 2000;43(6):837‐844. 10861878 10.1002/1522-2594(200006)43:6<837::aid-mrm9>3.0.co;2-o

[14] Wang S , Peng Y , Medved M , et al. Hybrid multidimensional T(2) and diffusion‐weighted MRI for prostate cancer detection. J Magn Reson Imaging. 2014;39(4):781‐788. 23908146 10.1002/jmri.24212 PMC4251798

[15] Sadinski M , Karczmar G , Peng Y , et al. Pilot study of the use of hybrid multidimensional T2‐weighted imaging‐DWI for the diagnosis of prostate cancer and evaluation of Gleason Score. AJR Am J Roentgenol. 2016;207(3):592‐598. 27352026 10.2214/AJR.15.15626 PMC5074540

[16] Chatterjee A , Bourne RM , Wang S , et al. Diagnosis of prostate cancer with noninvasive estimation of prostate tissue composition by using hybrid multidimensional MR imaging: a feasibility study. Radiology. 2018;287(3):864‐873. 29393821 10.1148/radiol.2018171130 PMC5978456

[17] Chatterjee A , Mercado C , Bourne RM , et al. Validation of prostate tissue composition by using hybrid multidimensional MRI: correlation with histologic findings. Radiology. 2022;302(2):368‐377. 34751615 10.1148/radiol.2021204459 PMC8805656

[18] Süli E , Mayers DF . Eigenvalues and eigenvectors of a symmetric matrix. An Introduction to Numerical Analysis. Cambridge University Press; 2003:133‐178.

[19] Bammer R . Basic principles of diffusion‐weighted imaging. Eur J Radiol. 2003;45(3):169‐184. 12595101 10.1016/s0720-048x(02)00303-0

[20] Gibbs P , Tozer DJ , Liney GP , Turnbull LW . Comparison of quantitative T2 mapping and diffusion‐weighted imaging in the normal and pathologic prostate. Magn Reson Med. 2001;46(6):1054‐1058. 11746568 10.1002/mrm.1298

[21] Gundogdu B , Pittman JM , Chatterjee A , et al. Directional and inter‐acquisition variability in diffusion‐weighted imaging and editing for restricted diffusion. Magn Reson Med. 2022;88(5):2298‐2310. 35861268 10.1002/mrm.29385 PMC9545544

[22] Szasz T , Lee G , Chatterjee A , et al. Physically implausible signals as a quantitative quality assessment metric in prostate diffusion‐weighted MR imaging. Abdom Radiol. 2022;47(7):2500‐2508. 10.1007/s00261-022-03542-0 35583823

